# Advances in the use of metal-free tetrapyrrolic macrocycles as catalysts

**DOI:** 10.3762/bjoc.20.257

**Published:** 2024-11-27

**Authors:** Mandeep K Chahal

**Affiliations:** 1 School of Chemistry and Forensic Science, University of Kent, Canterbury, CT2 7NH, UK

**Keywords:** calix[4]pyrroles, electrocatalysis, free-base porphyrins, organocatalysis, photocatalysis, tetrapyrrolic macrocycles

## Abstract

This review provides an overview of recent progress made in the field of catalysis using metal-free tetrapyrrolic macrocycles, focusing on calix[4]pyrroles, porphyrins and corroles, which are structurally related to porphyrins. Calix[4]pyrroles are versatile receptors in supramolecular chemistry while porphyrins are considered as ‘pigment of life’ due to their role in vital biological processes. Beyond their natural functions, synthetic porphyrins have been applied in various fields, including organometallic catalysis, dye-sensitized solar cells, sensing, artificial olfactory systems, photodynamic therapy (PDT), anticancer drugs, biochemical probes, and electrochemical devices. Relevant examples of these two pyrrolic macrocycles as metal-free organocatalysts, photocatalysts, and electrocatalysts are presented here. The effect of macrocyclic structural modifications such as their functionalization with different substituents, distortion from planarity, conformational flexibility and rigidity towards catalytic activity are presented, highlighting the potential of these two macrocycles as metal-free catalysts.

## Introduction

**Figure 1:**
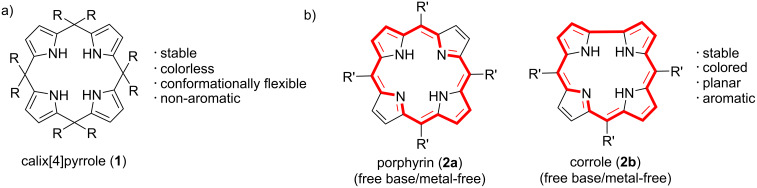
Chemical structures of the main tetrapyrrolic macrocycles studied in this review for their role as catalyst. a) calix[4]pyrrole **1** and b) porphyrin **2a** and corrole **2b**. The 18 π-electron aromatic system of porphyrin and corrole is highlighted by the red colour.

Tetrapyrrolic macrocycles are a class of cyclic compounds that contain four pyrrolic units in their ring. Examples of these are porphyrins, chlorins, porphyrazines, bacteriochlorins, corroles, calix[4]pyrroles, and phthalocyanines. One of the major differences between these pyrrolic macrocycles is how the adjacent pyrrole rings are connected. The most widely studied tetrapyrrolic macrocycles are typically π‐conjugated (aromatic) organic heterocyclic systems, excluding calix[4]pyrroles, which are colorless and non-aromatic, as well as norcorroles, isophlorins, and the 16π oxidized form of porphyrin that exhibits anti-aromatic character (Figure 1a). Calix[4]pyrroles possess a nonplanar structure and a high degree of conformational flexibility, allowing them to adopt four key conformations: 1,3-alternate, cone, partial cone, and 1,2-alternate [1–2]. Calix[4]pyrroles are one of the most studied hosts in supramolecular chemistry, finding use in applications of molecular recognition and extraction, drug delivery, ion transport and separation technology [3–8]. Conversely, porphyrins are connected via methine (=CH-) bridges, resulting in an 18 π-electron macrocyclic system affording macrocyclic planarity as well as unique photophysical and electrochemical properties (Figure 1b). While corroles share similarities with porphyrins, the direct linkage between their pyrrole units leads to a more contracted cavity compared to that of porphyrins. Similar to calix[4]pyrroles, synthetic metallo- and free-base (metal-free) porphyrins find various applications in the fields of medicine, energy, catalysis, molecular recognition, and supramolecular assemblies [9–13]. There are numerous examples of using metalloporphyrins as artificial photosynthesis models, enzyme mimics, and catalysts for various organic transformations, where a metal center acts as an active site [14–17]. However, metal-free (or free-base) macrocycles have not been explored as much in terms of catalysis, even though they are starting compounds for the preparation of their metallated analogues that are commonly used as catalysts.

In contrast with a calix[4]pyrrole macrocycle with four NHs (from four pyrrole units), a metal-free porphyrin macrocycle contains two Ns and two NHs (from two pyrrolenine and two pyrrole units), both of which can act as supramolecular H-bond donor and acceptors and can promote metal-free catalysis. Additionally, due to their synthetic versatility, these macrocycles can be further functionalized to add other binding sites required for substrate binding and/or promotion of the catalytic activity. Past studies have shown that modifying the porphyrin core with urea functionalities and amino acid substituents leads to the formation of ureaporphyrins, which significantly enhance sugar binding in non-polar solutions [18]. Similarly, Burns and co-workers reported di- and tetra-urea picket porphyrins highlighting, the impact of buried solvent molecules, such as DMSO, on the selectivity, affinity, and stoichiometry of anion binding [19]. Iron complexes of tetra-urea picket porphyrins further demonstrate how second-sphere interactions with a multipoint hydrogen-bonding pattern enhance CO_2_ reduction in organic solvents, improving stability, facilitating proton transfer, reducing energy barriers, and increasing selectivity [20]. Apart from advances in synthetic methodologies [2,21–23], the exploration of these macrocyclic catalysts is in a very nascent stage. In this review, the recent advancement in the field of metal-free macrocycles for catalysis will be summarized; mainly focused on porphyrins and calix[4]pyrroles and in the field of organocatalysis, photocatalysis, and electrocatalysis.

## Review

### 1 Metal-free tetrapyrrolic macrocycles as supramolecular organocatalysts

Supramolecular organocatalysis has recently attracted emerging attention as a green alternative to metal-based catalysis [24–26]. Organocatalysis using macrocyclic scaffolds such as crown ethers, cyclodextrins, cucurbiturils, and calixarenes has been extensively studied using both enzyme mimics and non-biomimetic systems, due to the presence of an internal cavity (binding sites) and nearby functional groups (catalytic sites) [27–29]. Tetrapyrrolic macrocycles contain an internal cavity with multiple inner –N/NH groups that function as hydrogen-bond donors and acceptors. Additionally, the nitrogen atoms in the pyrrole units of the porphyrin structure can also act as Lewis bases, capable of donating electron pairs. These properties enable tetrapyrrolic macrocycles to act as effective binding sites or catalytically active groups for a variety of substrates, making their use as supramolecular organocatalysts based on bifunctional activation mechanism (hydrogen-bonding/Lewis basicity) highly promising. At the same time, additional functional groups that are required for the catalysis can be easily installed on the periphery of tetrapyrrolic macrocycles using well established methodologies. This section focuses on examples where tetrapyrrolic macrocycles serve as organocatalysts. Firstly, various applications of calix[4]pyrroles as organocatalysts will be examined, followed by a discussion on organocatalysis using metal-free porphyrins.

#### 1.1 Calix[4]pyrrole macrocycles as organocatalysts

Calix[4]pyrroles act as versatile ligands in supramolecular chemistry and have been widely studied as binding hosts for various guests such as anions, ion pairs, or neutral compounds [4,30–31], ligands for p-block elements, as well as transition and rare-earth metals [32–33]. There are many comprehensive reviews covering these two areas along with the connection of these ligands to supramolecular and medicinal chemistry [34–36]. In addition, calix[4]pyrroles, due to the presence of four accessible inner NHs and well-defined binding pockets, offer a preorganized arrangement of functional groups as a suitable microenvironment for organocatalysis.

**Figure 2:**
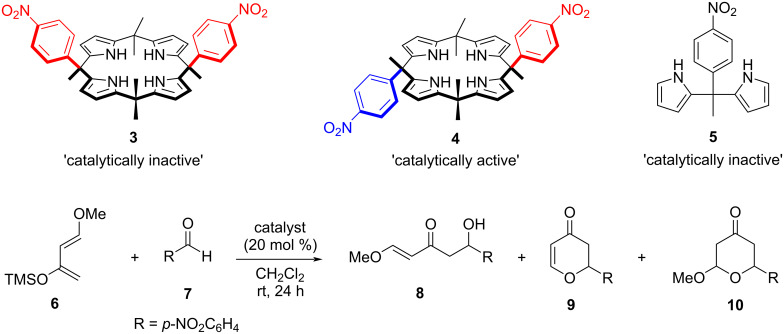
Calix[4]pyrroles **3** and **4** and an their acyclic analogue **5** used for the transformation of Danishefsky's diene **6** and *p*-nitrobenzaldehyde (**7**) to the respective Mukayama aldol (**8**) and products of a hetero-Diels–Alder reaction (**9** and **10**); *p*-nitrophenyl units in red and blue, pointing upwards and downwards, respectively. Adapted from [37].

In 2008, Kohnke, Soriente and co-workers first reported [37] the H-bonding organocatalytic activity of calix[4]pyrrole derivatives **3** and **4** and acyclic dipyrromethane **5** for the hetero-Diels–Alder reaction of Danishefsky's diene **6** with *p*-nitrobenzaldehyde (**7**, Figure 2). The reaction can provide three products depending on the reaction conditions; either a Mukaiyama aldol (**8**) or products of Diels–Alder cycloaddition (**9** and **10**). Out of the three screened catalysts, only calix[4]pyrrole α,β-isomer **4** was found to be catalytically active providing a 57% conversion to **10**, suggesting a concerted cycloaddition mechanism. Calix[4]pyrrole α,α-isomer **3** and dipyrromethane **5** were catalytically inactive. The authors concluded that the catalytic inactivity of **3** is caused by the parallel orientation of *p*-nitrophenyl units, due to the shielding of the bound aldehyde substrate from the incoming diene. The catalytic inactivity of **5** demonstrated the requirement of macrocyclic character for the potential catalysts.

**Figure 3:**
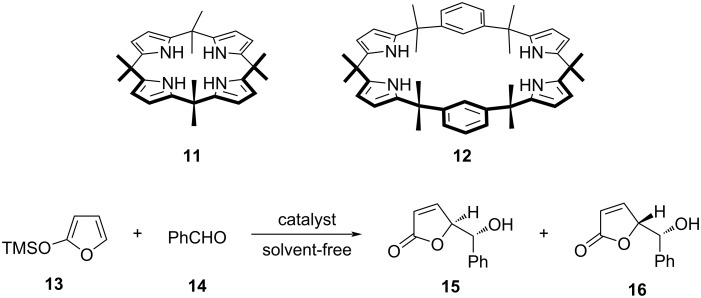
Calixpyrrole-based organocatalysts **11** and **12** for the diastereoselective addition reaction of TMSOF **13** and benzaldehyde (**14**) providing the respective erythro **15** and threo **16** aldol product. Adapted from [38].

Later in 2009, the same group reported an organocatalyzed diastereoselective aldol addition of furan-based silyloxydiene synthons to a variety of achiral aldehydes using four different calix[4]pyrrole macrocycles (**3**, **4**, **11**, and **12**) as organocatalysts (Figure 3) [38]. These calixpyrrole macrocycles acted as hydrogen-bond donors, activating substrate aldehydes through hydrogen-bonding interactions and accelerating aldol reactions. In the absence of a catalyst, no reaction between 2-(trimethylsilyloxy)furan (TMSOF, **13**) and benzaldehyde (**14**) was observed**,** whereas all the tested macrocyclic compounds were found catalytically active, with **11** being the most efficient providing erythro/threo (**15**/**16**) aldol products with up to 82% yield in a 70:30 diastereoisomeric ratio.

**Figure 4:**
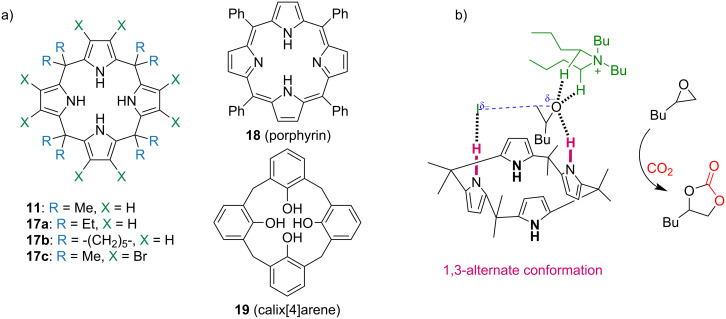
(a) Chemical structures of macrocyclic organocatalysts used for the synthesis of cyclic carbonates from epoxides and CO_2_; (b) Structure of the DFT-calculated transition state in the **11**/TBAI-catalysed reaction of 1,2-epoxyhexane **20** with CO_2_. Adapted from [39].

A decade after, Ema, Maeda and co-workers investigated using of calix[4]pyrrole macrocyclic organocatalysts for the synthesis of cyclic carbonates **21** from epoxides **20** (1,2-epoxyhexane) and CO_2_ [39]. For this purpose, they used three different types of macrocycles: calix[4]pyrroles **11**, **17a**–**c**, porphyrin **18**, and calix[4]arene **19** (Figure 4a). Despite the presence of –OH and –NH binding sites, both calix[4]arene **19** and porphyrin **18** showed only a negligible activity compared to calix[4]pyrroles (**11**, **17a**–**c**), which provided, with TBAI as a co-catalyst, up to 74% yields (Table 1). The inactivity of porphyrin **18** was attributed to the inaccessibility of the inner core imine due to its planar structure. The mechanism of the epoxide ring-opening reaction was elucidated by DFT calculations, which suggested that the macrocycle adopts a 1,3-alternate conformation and binds simultaneously to the epoxide O-atom and iodide anion via (NH···O and NH···I) hydrogen-bonding interactions. The TBA countercation is bound to the O-atom of the epoxide ring with hydrogen bonds and is situated away from the I^−^ anion. This crucial transition state stabilizes the anionic species generated during the reaction pathway and facilitates a backside attack of I^−^ on the epoxide thus resulting in the initial ring opening (Figure 4b).

**Table 1:** Organocatalytic activity of calix[4]pyrrole macrocycles **11**, **17a**–**c** for CO_2_ insertion into the epoxide **20** leading to the cyclic carbonate **21**.

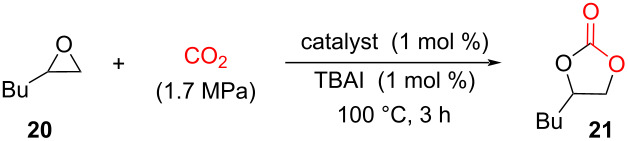	

Catalyst	Yield (%)

–	21
**11**	74
**11** ^a^	0
**17a**	28
**17b**	40
**17c**	41
**18** ^b^	9
**19** ^b^	9

^a^Without TBAI; ^b^cat. (0.5 mol %), TBAI (1 equiv to cat.), 75 °C, 6 h.

**Figure 5:**
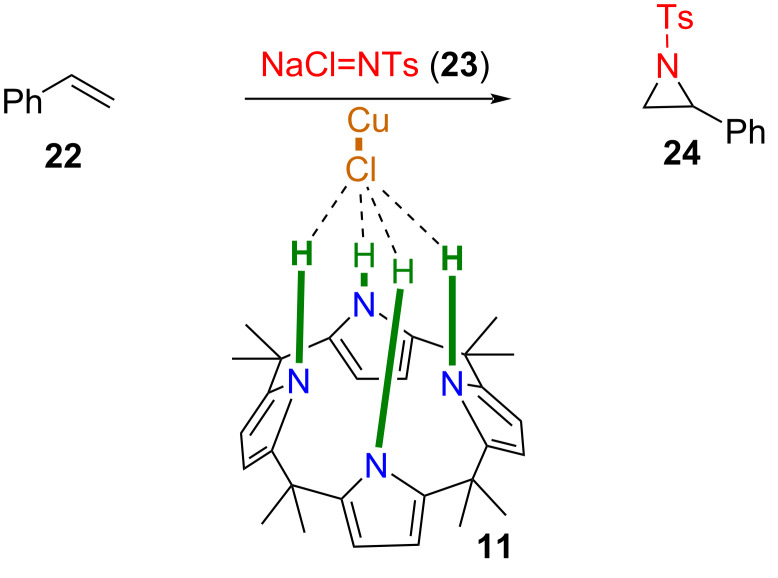
Cuprous chloride-catalyzed aziridination of styrene (**22**) by chloramine-T (**23**) providing 1-tosyl-2-phenylaziridine (**24**) (top); suggested structure of a catalytically active intermediate of CuCl and calix[4]pyrrole **11** (below). Adapted from [40].

Apart from acting as an organocatalyst, calix[4]pyrrole **11** has been used for the promotion of cuprous chloride-catalyzed aziridination of styrene (**22**) by chloramine-T (**23**, NaCl=NTs) as a source of nitrene in acetonitrile (Figure 5) [40]. No aziridine product was formed either without any source of copper or in the presence of a different copper salt, such as CuCl, CuCl_2_·2H_2_O, or CuOTf. Calix[4]pyrrole itself is catalytically inactive, but the mixture of CuCl (7 mol %) and calix[4]pyrrole (14 mol %) resulted in a 74% yield of 1-tosyl-2-phenylaziridine (**24**). Considering the significant shift (from 7.48 to 9.98) in the N–H signal of calix[4]pyrrole after the addition of CuCl, the authors suggested that calix[4]pyrrole activates the Cu–Cl bond via chloride···calixpyrrole (N–H···Cl) hydrogen-bonding interactions toward the formation of the nitrene intermediate from chloramine-T (NaCl=NTs). Additionally, calix[4]pyrrole served as a phase-transfer catalyst in this reaction. Since chloramine-T had low solubility in acetonitrile, calix[4]pyrrole enhanced its solubility, contributing to its indirect activation. Various control experiments, such as using CuI with and without calix[4]pyrrole and using dipyrromethane as another potential co-catalyst, have confirmed the role of calix[4]pyrrole as a promoter.

Recently, Ballester and co-workers reported on the preparation of an octapyridinium-based water-soluble superaryl-extended calix[4]pyrrole molecular container and used it as a capsule for desymmetrization reactions [41], where the reported compound acts both as sequestering and supramolecular protecting group.

All of the examples mentioned above indicate that calix[4]pyrroles can be used as organocatalysts. Despite major advancements in synthetic methodologies to synthesize functionalized calix[4]pyrrole macrocycles, not much progress has been done in this area in recent years. One of the major challenges of using calix[4]pyrroles as catalysts may be related to their conformational flexibility, that leads to less preorganized binding and catalytic sites. Calix[4]pyrroles in solution exist in four-different conformations (cone, partial cone, 1,3-alternate, and 1,2-alternate); this macrocyclic flexibility arises due to the sp^3^-linkage between the pyrrole units that allows their inversion through the plane of the macrocycle and could inhibit the organocatalytic activity.

#### 1.2 Porphyrin macrocycles as organocatalysts

Porphyrins can coordinate almost any metal from the periodic table [42–43], they offer high functional versatility [44], and many of these resulting metal complexes are catalytically active [45–47]. These synthetic metalloporphyrins take inspiration from biological systems, such as hemes (iron complexes), chlorophylls (magnesium complexes), and vitamin B_12_ (cobalt complex).

Contrary to metalloporphyrins that are easily accessible for the incoming substrates, pyrrole –N/NH moieties inside the core of metal-free porphyrins are mostly hidden and unavailable for any kind of intermolecular hydrogen-bonding interactions or molecular recognition as they are 'shielded' by the planar macrocyclic system [48]. Therefore, most of the work involving metal-free porphyrins is limited to investigations on N–H tautomerization and protonation–deprotonation studies [49–52]. However, there are several chemical tools to convert the planar geometry of porphyrins to nonplanar, such as functionalization at β- and *meso*-positions, N-alkylation, arylation or protonation, interruption of the conjugated system, reduction/oxidation of the macrocycle and/or strapping of the macrocycle via covalent linkage of the *meso*- or β-pyrrole positions [22,53–57]. These alternations can significantly affect the optical and electronic properties, as well as the reactivity of porphyrins, mainly introducing non-planarity with easier access to the inner pyrrolic –NHs and –N-lone pairs. Additionally, these alterations potentially increase Lewis basicity that further improves interactions with substrates. Changes in the reduction or oxidation state can alter redox behavior, thereby affecting catalytic activity. For example, it has been reported that 2,3,5,7,8,10,12,13,15,17,18,20-dodecasubstituted free-base porphyrins and their mono/diprotonated derivatives are highly distorted with a good access to the pyrrolic N/N–H moieties [58–60]. Overall, these alterations provide a versatile toolkit for tailoring porphyrin properties for various applications.

**Figure 6:**
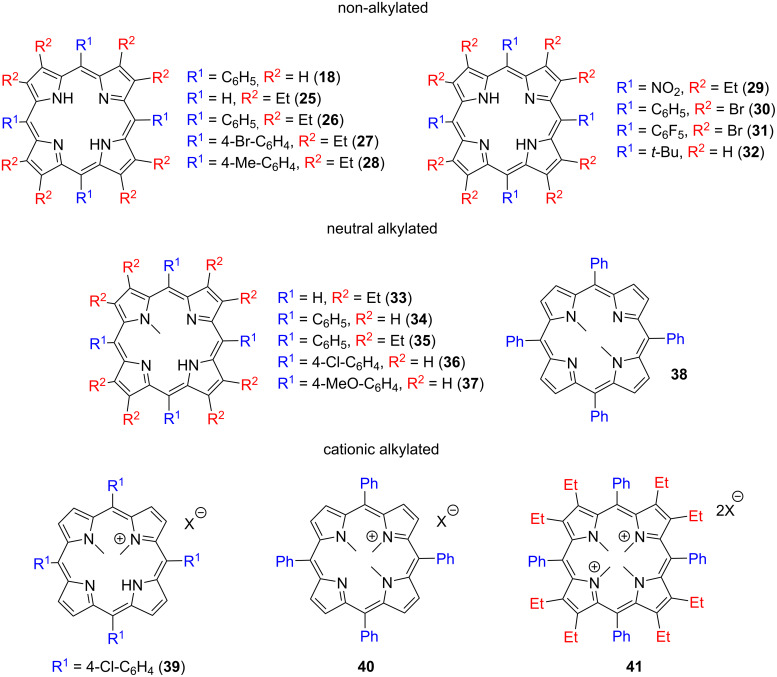
Chemical structures of the various porphyrin macrocycles (**18**, **25**–**41**) screened as potential catalysts of the sulfa-Michael addition reaction between thiol **42** and phenyl vinyl sulfone (**43**). Adapted from [61].

**Figure 7:**
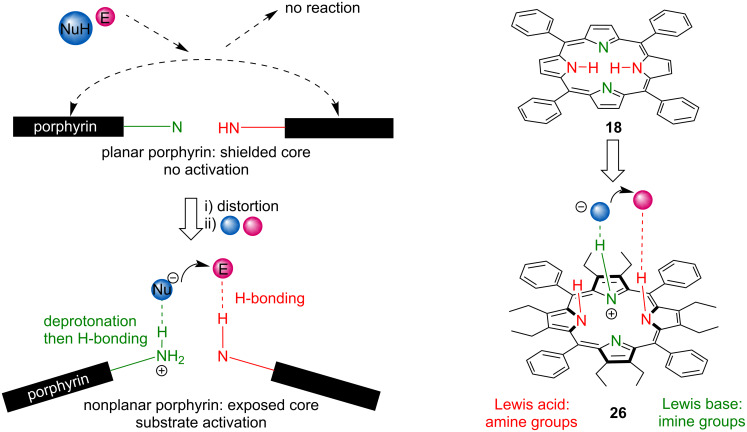
Organocatalytic activity of distorted porphyrins explored by Senge and co-workers. Planar macrocycle **18** is unable to bind/activate small molecules. With the increase in distortion, the macrocycle’s core becomes available for intermolecular interactions. Figure 7 was adapted from [62], M. Kielmann et al., ‘’Incremental Introduction of Organocatalytic Activity into Conformationally Engineered Porphyrins’’, Eur. J. Org. Chem., with permission from John Wiley and Sons. Copyright © 2019 WILEY-VCH Verlag GmbH & Co. KGaA, Weinheim. This content is not subject to CC BY 4.0.

In 2017, Senge and co-workers, reported the first example of using metal-free tetrapyrrolic porphyrins as bifunctional organocatalysts, confirming that the distortion/nonplanarity of the macrocycle and the resulting availability of pyrrolic protons is necessary for catalytic activity [61]. A set of 18 different metal-free porphyrins (non-alkylated, neutral alkylated, and cationic alkylated) with varying degrees of distortion from planarity as well as different electronic properties (**18**, **25**–**41**, Figure 6) were screened as catalysts for the sulfa-Michael addition of *tert*-butyl benzylmercaptan **42** to phenyl vinyl sulfone (**43**). Without the addition of a porphyrin, no product was formed. Among the non-alkylated porphyrins (**18**, **25**–**32**) only the ones containing ethyl groups at the β-position and C_6_H_5_ or 4-Me-C_6_H_4_ at the *meso*-position (**26** and **28**) were catalytically active, giving more than 98% conversion, whereas the planar derivatives; H_2_OEP (2,3,7,8,12,13,17,18-octaethylporphyrin (**25**)), H_2_TPP (5,10,25,20-tetraphenylporphyrin (**18**)) and all the compounds with electron-withdrawing substituents at the *meso*- and/or β-positions and highly saddle-distorted geometry (**27**, **29**–**31**) are inactive (Table 2). Mono-N-alkylation of the macrocycles resulted in a slight improvement of activity giving up to 50–62% conversion for **34** and **37**, both of which are alkylated versions of an inactive tetraarylporphyrin **18**, by increasing the porphyrin basicity and distortion. On the other hand, di-N-alkylation of **18** (providing compound **38**) reduced the catalytic activity to only 5% conversion. The authors also screened cationic N-alkylated macrocycles (**39**–**41**) and found that only **39** with one remaining –NH group is catalytically active while both tri- and tetraalkylated analogues **40** and **41**, without an –NH unit, are not. Further, the authors performed ^1^H NMR experiments with a different substrate:macrocycle ratio and suggested a bifunctional reaction mechanism involving both inner amine and imine groups (Figure 7).

**Table 2:** Organocatalytic activity of porphyrins **18**, **25**–**41** for the synthesis of **44** from **42** and **43**.

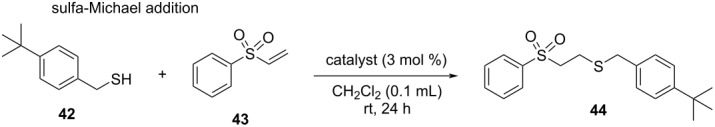	

Catalyst	Yield (%)^a^

–	0
**18**, **25**, **27**, **29-32**	0
**26**	>98
**28**	>98
**33**	<5
**34**	50
**35**	>98
**36**	3
**37**	62
**38**	5
**39**	>98
**40**, **41**	0

^a^Determined by ^1^H NMR spectroscopy using an internal standard.

**Figure 8:**
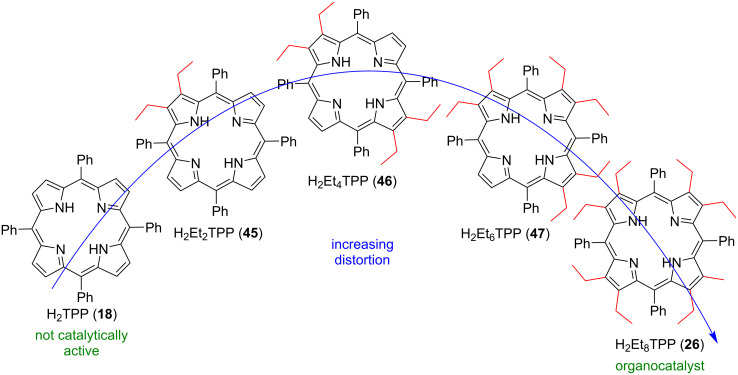
Chemical structures of H_2_Et_x_TPP (*x* = 0, 2, 4, 6, 8) compounds with incrementally increasing nonplanarity used to explore effect of electronic and steric factors on the organocatalytic activity. Figure 8 was adapted from [62], M. Kielmann et al., ‘’Incremental Introduction of Organocatalytic Activity into Conformationally Engineered Porphyrins’’, Eur. J. Org. Chem., with permission from John Wiley and Sons. Copyright © 2019 WILEY-VCH Verlag GmbH & Co. KGaA, Weinheim. This content is not subject to CC BY 4.0.

Later the same group synthesized a series of five macrocycles derived from tetraphenylporphyrin (H_2_TPP) with a different number of ethyl substituents at the β-positions; H_2_Et_x_TPPs (*x* = 0, 2, 4, 6, 8; **18**, **45**–**47**, **26**, Figure 8) to explore the effect of electronic and steric factors on the organocatalytic performance in the same reaction as before (Table 2) [62]. Among the tested compounds, the highly nonplanar macrocycle **26** with a good accessibility of both pyrrolic –N/N–H moieties turned out to be the best candidate, giving an 80% conversion yield, whereas the other compounds (**18**, **45**–**47**) provided only a trace amount of the product.

**Figure 9:**
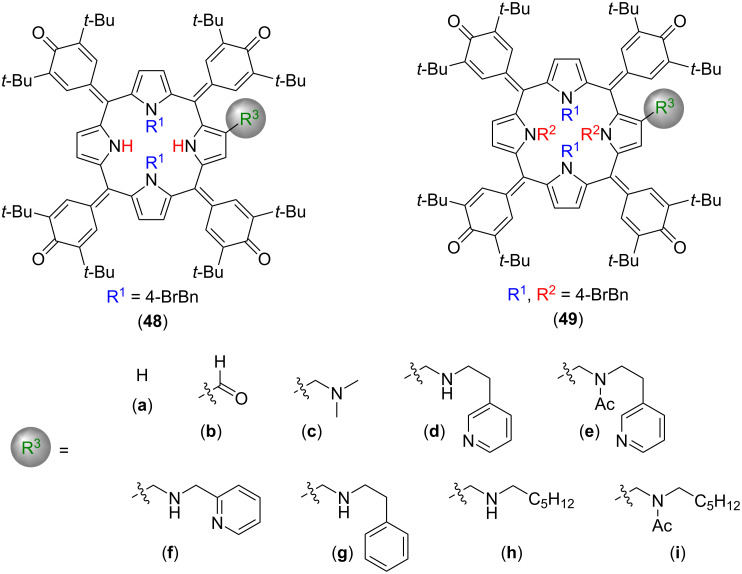
Chemical structures of OxP macrocycles tested as potential organocatalysts for the conjugate addition of 2,4-pentanedione (**51**) to β-nitrostyrene (**50**). Adapted from [63].

Considering the nonplanarity of a metal-free porphyrin as an essential requirement for its catalytic activity, Hill and co-workers explored the use of oxidized porphyrin macrocycles, also known as oxoporphyrinogens (OxPs), **48** and **49** for the 1,4-conjugate addition (Michael addition) of 2,4-pentanedione (**51**) to β-nitrostyrene (**50**) (Figure 9) [63]. The OxP-macrocycles turned out to combine the advantages of porphyrins and calix[4]pyrroles. Due to their nonplanar geometry, OxPs have easily accessible inner –NH groups, similarly to calix[4]pyrroles, and at the same time their conformation is rigid due to the presence of sp^2^-hybridized carbon bridges between the pyrrole units and alkyl groups on two of the inner N atoms of the macrocycle [64–66]. Among the OxP derivatives tested for organocatalysis (**48a**–**i** and **49a**–**i**), only N-dialkylated ones with secondary amine side arm (**48d**, **48g**, **h**) were catalytically active for Michael additions, providing 60–71% yields (Table 3), whereas tetraalkylated analogues (**49a**–**g**) and dialkylated OxPs without a secondary amine side arm (**48a**–**c**, **48e** and **48i**) were not. Based on these results, the authors have concluded that both the presence of hydrogen-bond donor moieties (pyrrolic –NH groups) and a basic β-substituent are necessary to make the compound catalytically active. Further, authors have performed ^1^H NMR binding and kinetic studies and suggested that the reaction mechanism involves a simultaneous activation of both substrates via hydrogen-bonding interactions. Additionally, these macrocycles showed excellent activity for sulfa-Michael additions, as well as a moderate activity for Henry and aza-Henry reactions. These results are consistent with the observation reported by Senge and co-workers, establishing that nonplanarity and the presence of both basic Ns and NHs capable of hydrogen bonding are necessary for making metal-free tetrapyrrolic macrocycles catalytically active.

**Table 3:** Organocatalytic activity of tetrapyrrolic macrocycles **48a**–**i** and **49a**–**i** for the synthesis of **52** (Michael addition product) from **50** and **51**.

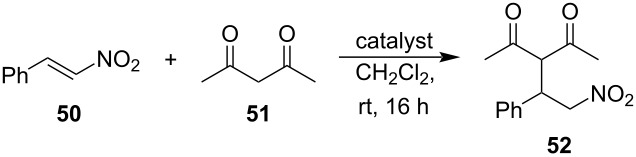		

Catalyst	Catalyst loading (mol %)	Conversion (%)

–	–	0
**48a, 48b, 49a, 49b**	1.0	0
**48c**	1.0	10
**48d**	0.5	71
**49d**, **48e**, **49h**, **48i**	0.5	0
**48f**	0.5	<5
**48g**	0.5	63
**48h**	0.5	60

**Figure 10:**
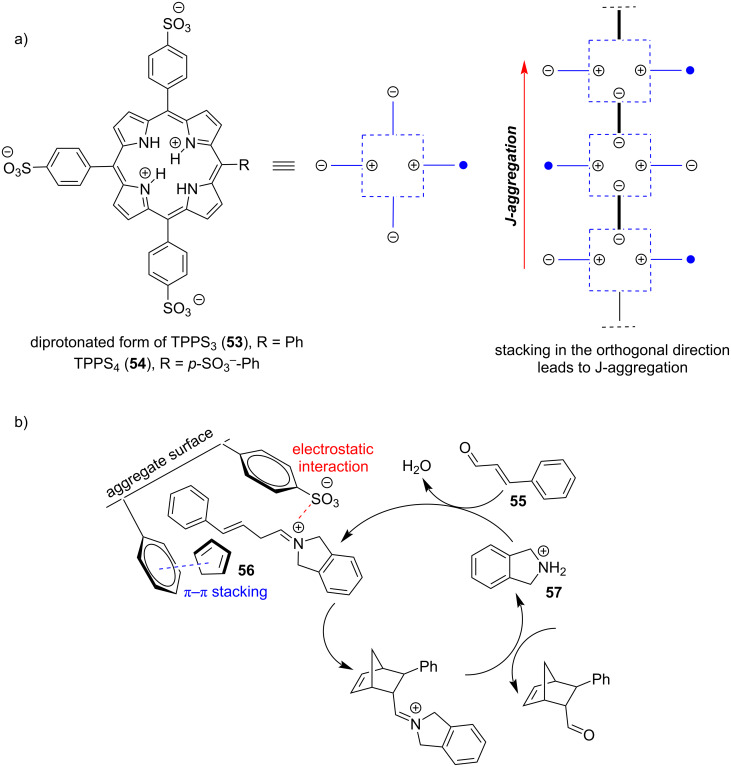
a) Fundamental structure of the J-aggregates of diprotonated TPPS_3_ **53** and b) its use as a catalyst of Diels–Alder reaction. Figure 10 was reproduced from [67] (© 2018 A. Arlegui et al., published by MDPI, distributed under the terms of the Creative Commons Attribution 4.0 International, https://creativecommons.org/licenses/by/4.0).

An alternative approach for making metal-free porphyrins catalytically active is based on using amphiphilic macrocycles and their aggregates. Moyano, Crusats and co-workers have done an extensive work on the development of supramolecular organocatalysts containing an amphiphilic metal-free porphyrin *meso*-(4-sulfonatophenyl)porphyrin and its J-aggregates [67–70]. In acidic (pH < 4.8) aqueous solutions, the central pyrroleninic core of the porphyrin is diprotonated, which induces the formation of supramolecular aggregates, stabilized by ion-pair contacts (electrostatic interactions) between the cationic porphyrin centers and anionic sulfonate groups of the periphery (Figure 10a). In 2018, the group reported heterogeneous catalysis of Diels–Alder reaction in aqueous environment catalyzed by TPPS_3_ **53** supramolecular aggregates [67]. The Diels–Alder reaction between cinnamaldehyde (**55**) and cyclopentadiene (**56**) proceeds via iminium activation by the zwitterionic hetero-aggregates derived from TPPS_3_ molecules **53** and a cyclic secondary amine **57**. They have hypothesized that the organocatalytic activity of the aggregates is based on two types of interactions, i.e., electrostatic interactions of α,β-unsaturated iminium cations derived from cinnamaldehyde and the cyclic secondary amine with anionic sulfonate groups and π–π interactions between phenyl groups and cyclopentadiene. Due to the presence of both types of moieties on the aggregate surface, the two reacting species can get into proximity and form the desired product (Figure 10b).

**Figure 11:**
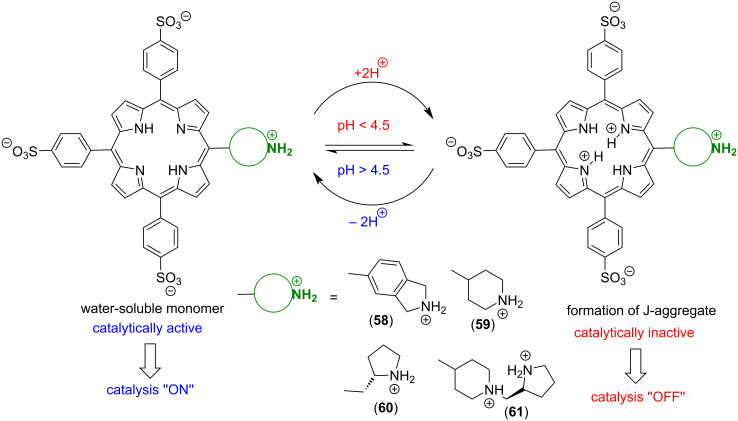
Chemical structures of amphiphilic porphyrin macrocycles used as pH-switchable catalysts based on in situ aggregation/dissociation. Adapted from [68–69].

Later, an analogous system was used for catalysis of an asymmetrical Diels–Alder reaction. Although *meso*-tetrakis(4-sulfonatophenyl)porphyrin (TPPS_4_, **54**) is an achiral molecule, the respective J-aggregates reveal supramolecular chirality caused by spontaneous mirror symmetry breaking (SMSB) during the aggregation process in an aqueous acidic solution. Using of these aggregates led to enantiomeric excess (ee) up to 5.5% [70]. Related catalytic systems based on amphiphilic 5-(cyclic-secondary-amine)-10,15,20-tris(4-sulfonatophenyl)porphyrin macrocycles **58**–**61** act as switchable organocatalysts for Michael and aldol reactions in water [68–69]. The macrocycles **58**–**61** containing different chiral or achiral cyclic secondary amine moieties oscillate between the aggregated and non-aggregated state depending on pH (Figure 11). The diprotonated species generated at lower pH forms supramolecular aggregates whereas the metal-free macrocycle is unable to aggregate and remains in the solution as a monomer. Since the aggregates were found catalytically inactive, while the monomers in the solution were active, the system acts as a pH-switchable ‘ON–OFF’ organocatalyst. In the case of the enamine-mediated addition of cyclohexanone (**62**) to 4-nitrobenzaldehyde (**7**), using 10 mol % of **58** provided up to 99% yield with a 93:7 ratio of the *anti*:*syn* aldol product (**63a**:**63b**) and no enantioselectivity at pH 6.7, whereas at pH 3.6 the catalyst was completely inactive (Table 4). Although the supramolecular system composed of a porphyrin macrocycle and a secondary amine organocatalyst operated through the reversible formation of covalent enamine intermediates, it also leveraged the supramolecular behavior of the porphyrinic component. In acidic aqueous media, the porphyrin macrocycle formed supramolecular H- and J-aggregates stabilized by hydrophobic interactions between the π-systems of the aromatic regions, along with electrostatic and hydrogen-bonding interactions. This behavior not only allowed for the selective activation and deactivation of organocatalytic activity but also facilitated efficient catalyst recovery at the end of the catalytic reaction. Notably, control experiments supported the hypothesis that the reaction would work in acidic environment using catalysts insensitive to pH-induced aggregation.

**Table 4:** Organocatalytic activity of amphiphilic porphyrins **58**–**61** for aqueous aldol reaction of cyclohexanone (**62**) with 4-nitrobenzaldehyde (**7**).

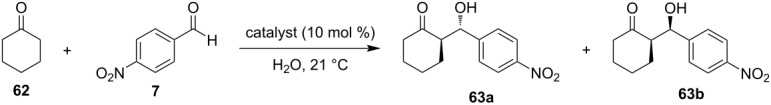				

Catalyst	pH	Yield (%)^a^	**63a**:**63b** (dr)^b^	% ee^c^

**58**	3.6	0	–	–
**58**	6.7	99	93:7	–
**59**	3.6	0	–	–
**59**	4.0	0	–	–
**59**	6.7	100	66:34	–
**60**	6.7	96	63:37	1.9 (2S)/0
**61**	6.7	89	70:30	16.7 (2S)/11.8 (2S)

^a^Isolated yield of racemic aldol (**63a** + **63b**) after chromatographic purification. ^b^Determined by ^1^H NMR (400 MHz) of the crude reaction mixture before chromatographic purification. ^c^Determined by chiral HPLC for **63a** (*anti*) and **63b** (*syn*), respectively.

In the same aldol reaction, using of macrocycles **60** and **61** containing chiral secondary amine moieties provided not only good yields, but also good diastereoselectivities; chiral HPLC analysis of the aldol product mixture showed that the reaction mixture contained only a negligible amount (1.9% ee) of the *anti*-isomer **63a** and *syn*-diastereomer **63b** was obtained in the racemic form when **60** was used as an organocatalyst. On the other hand, when using **61**, both diastereomers were obtained in optically active form with 16.7% ee for **63a** and 11.8% ee for **63b**, respectively (Table 4). The pH-induced aggregation does not only enable to control the catalytic activity, but it also allows a straightforward separation and recovery of the catalyst from the reaction mixture by acidification and centrifugation.

In the same way as a calix[4]pyrrole was used as organocatalyst for cyclic carbonate synthesis from epoxide and CO_2_, as discussed in section 1.1, Gallo and co-workers investigated the organocatalytic activity of porphyrin/TBACl binary catalytic systems for the regioselective cycloaddition of CO_2_ to *N*-alkyl/arylaziridines providing *N*-alkyl/aryloxazolidin-2-ones [71–73].

**Figure 12:**
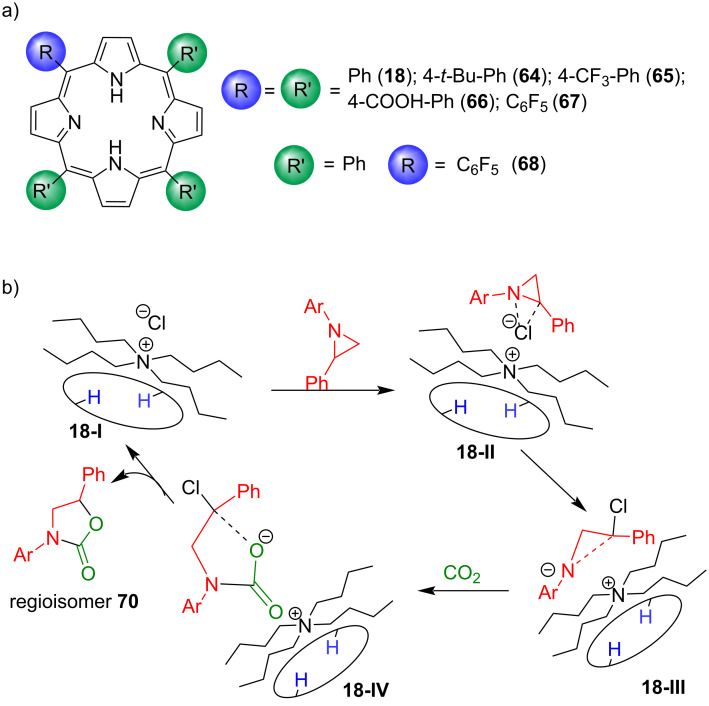
a) Chemical structures of porphyrin macrocycles for the cycloaddition of CO_2_ to N-alkyl/arylaziridines and b) proposed mechanism for the synthesis of N-aryloxazolidin-2-one **70** using porphyrin macrocycles as catalysts. Adapted from [71–73].

They used seven different planar tetraarylporphyrin organocatalysts; **H_2_TPP** (tetraphenylporphyrin, **18**)**_,_ H_2_4-*t*-BuTPP** (tetrakis(4-*tert*-butylphenyl)porphyrin, **64**), **H_2_4-CF_3_TPP** (tetrakis(4-trifluoromethylphenyl)porphyrin, **65**), **H_2_4-COOHTPP** (tetrakis(4-carboxyphenyl)porphyrin, **66**), **H_2_F_20_TPP** (*meso*-tetrakis(pentafluorophenyl)porphyrin, **67**), **H_2_F_5_TPP** (5-(pentafluorophenyl)-10,15,20-triphenylporphyrin, **68**) and **H_2_OEP** (octaethylporphyrin, **25**) (Figure 12a), all of which were found catalytically active under optimized reaction conditions (catalyst/TBACl/aziridine 1:5:100 and 1.2 CO_2_ MPa at 125 °C) [71]. Out of all the used macrocycles, the unsubstituted **H_2_TPP** (**18**)/TBACl system turned out to be the best, giving up to 95% yield for the both *N*-alkyl/arylaziridine substrates with regioisomeric ratios up to 95:5 (**70b**:**71b**) for R = *n*-Bu and 87:13 (**70a**:**71a**) for R = 3,5-(CF_3_)_2_C_6_H_3_. It was found out that increasing the steric features on the catalyst skeleton resulted in only marginally lower yields, suggesting that the electronic and steric features of the employed porphyrin have only a limited influence on the catalytic performances (Table 5). DFT calculations predicted that the catalytically active species is the adduct of porphyrin and TBACl (**18-I**), which forms an activated complex (**18-II**) with the substrate followed by a ring-opening nucleophilic attack of Cl^−^. The electron-rich nitrogen atom in **18-III** further activates electrophilic CO_2_, leading to the formation of **18-IV**. The negatively charged oxygen in **18-IV** is then responsible for removing the chloride atom leading to the major isomer as a final product.

**Table 5:** Organocatalytic activity of planar porphyrins (**18**, **64**–**68**, and **25**)/TBACl catalytic systems for the synthesis of oxazolidin-2-ones **70** and **71**.

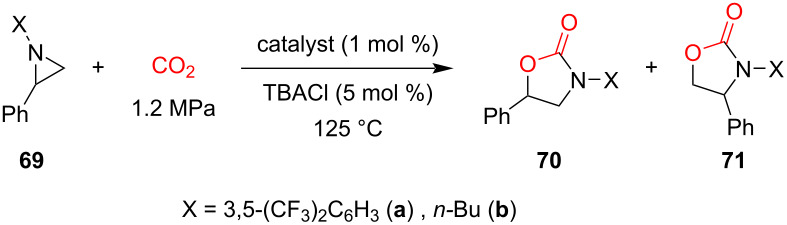		

Catalyst	Yield (%) (**70a:71a**)	Yield (%) (**70b:71b**)

**18** (H_2_TPP)	95 (**87:13**)	95 (**95:5**)
**64** (H_2_4-*t*-BuTPP)	69 (**83:17**)	94 (**91:9**)
**65** (H_2_4-CF_3_TPP)	61 (**85:15**)	80 (**92:8**)
**66** (H_2_4-COOHTPP)	84 (**86:14**)	99 (**86:14**)
**67** (H_2_F_20_TPP)	43 (**84:16**)	74 (**89:11**)
**68** (H_2_F_5_TPP)	69 (**87:13**)	76 (**88:12**)
**25** (H_2_OEP)	63 (**84:16**)	85 (**93:7**)

The results of this study suggest that nonplanarity or distortion of the tetrapyrrolic macrocyclic core is not a necessary condition to make them organocatalytically active. Even planar porphyrin macrocycles in combination with ammonium salts can act as effective catalysts. Later in 2023, the same group reported the use of protonated H_2_TPP **18** as a bifunctional metal-free porphyrin catalyst for the synthesis of *N*-alkyloxazolidinones, eliminating the need for any Lewis base or additives [74]. This represented a significant advancement over their previously reported work. They used six different protonated porphyrins as catalysts: TPPH_4_X_2_ (**18a**, X = Cl; **18b**, X = Br; **18c**, X = I), and TPPH_4_(RCOO)_2_ (**18d**, R = CF_3_; **18e**, R = ClCH_2_; **18f**, R = Cl_2_CH), all of them were synthesized quantitatively from commercially available tetraphenylporphyrin, H_2_TPP (**18**). They screened these catalysts for the synthesis of *N*-butyl-phenyloxazolidin-2-one **70b** from **69b** using 1% of catalyst under 1.2 MPa of CO_2_ pressure. The reactions were conducted at 100 °C for 6 hours in dichloroethane (DCE). All catalysts demonstrated regioselectivities of 95:5 (**70b**:**71b**) with 100% selectivity and good conversions (60% for **18a**, 84% for **18b**, 100% for **18c**, 8% for **18d**, 27% for **18e**, and 8% for **18f**), irrespective of the nature of anion. Additionally, they performed DFT studies to elucidate the mechanism of CO_2_ cycloaddition to aziridines using a metal-free protonated porphyrin macrocycle and found that the catalytic cycle started with simultaneous activation of both CO_2_ and *N*-butyl-2-phenylaziridine (**69b**).

The main strategies used in metal-free porphyrin organocatalysis can be summarized in the following statements: (1) using highly distorted nonplanar macrocyclic systems with an easy access to inner –NHs and basic imine moieties (by Senge, Hill, and co-workers [61–63]), (2) using monomeric and aggregated forms of achiral/chiral planar amphiphilic porphyrin systems (by Moyano, Crusats, and co-workers [67–70]), and (3), using planar porphyrin macrocycles in combination with ammonium salts as co-catalysts as well as protonated porphyrins (by Gallo and co-workers [71–74]). Hence, considering the wider functionalities associated with porphyrin macrocycles, both synthetic and found in nature, and their ability to act as organocatalysts, metal-free porphyrin macrocycles have a potential to be excellent candidates for green, cost-effective catalysts of various organic transformations including asymmetric synthesis.

### 2 Metal-free tetrapyrrolic macrocycles as photoredox catalysts

Supramolecular photocatalysis using different metal-free macrocyclic hosts, including cyclodextrins, cucurbiturils, porphyrins, and calixarenes has been extensively explored due to their unique characteristics, such as ease of modification, presence of hydrophobic cavities, and ability of specific guest recognition via noncovalent interactions [75–78]. In general, macrocycles provide an appropriate platform for the design and construction of supramolecular catalytic systems, since macrocycles can act both as stabilizers and electron transporters in supramolecular systems. This section covers advancements in the field of metal-free macrocyclic photocatalysis, with a focus on porphyrin macrocycles, since calix[4]pyrroles do not act as photosensitizer. As the field of metal-free porphyrins as photoredox catalysts is still in its early stages, there are only a few examples present in the literature. This section also includes the first example of photoredox catalysis utilizing corroles, another tetrapyrrolic photosensitizers.

**Figure 13:**
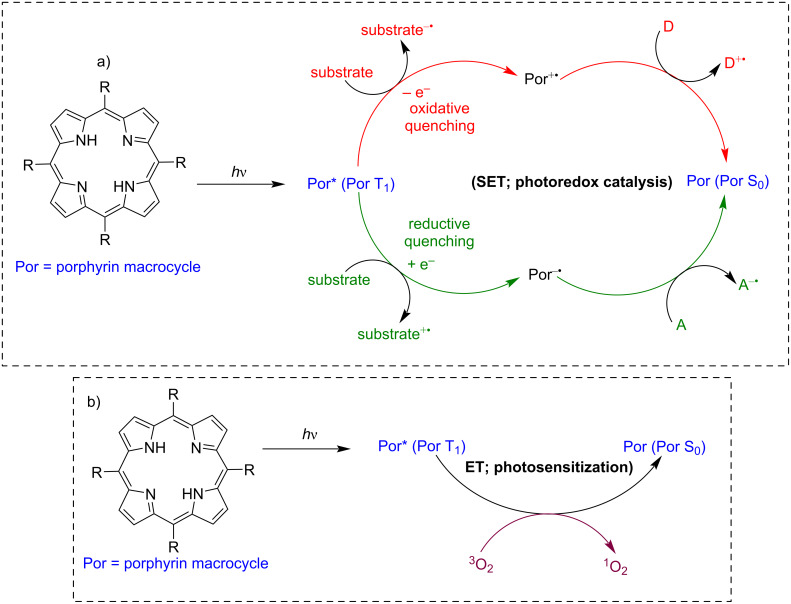
Electron and energy-transfer processes typical for excited porphyrin molecules (Por = porphyrin macrocycle). a) Single-electron transfer and b) energy transfer. Adapted from [84–86].

Porphyrins are well-known photosensitizers widely studied for their use in photobiology. Their extensive aromatic system enables them to absorb significant amounts of visible light photons, which allows them to reach an excited state. The excited porphyrin molecule is likely to undergo energy transfer (ET; photosensitization) or single-electron transfer (SET; photoredox catalysis) to substrate molecules (Figure 13). In photochemistry, porphyrins are mainly used for the generation of singlet oxygen (^1^O_2_) or other reactive oxygen species. Porphyrins in the triplet excited state can relax to the ground state by transferring energy to molecular oxygen (triplet state) forming ^1^O_2_ (Figure 13b) [67]. Photosensitized singlet oxygen (^1^O_2_) finds many applications in photochemistry and photobiology, e.g., for wastewater treatment, fine chemical synthesis, and photodynamic therapy (PDT) [79–83].

**Figure 14:**
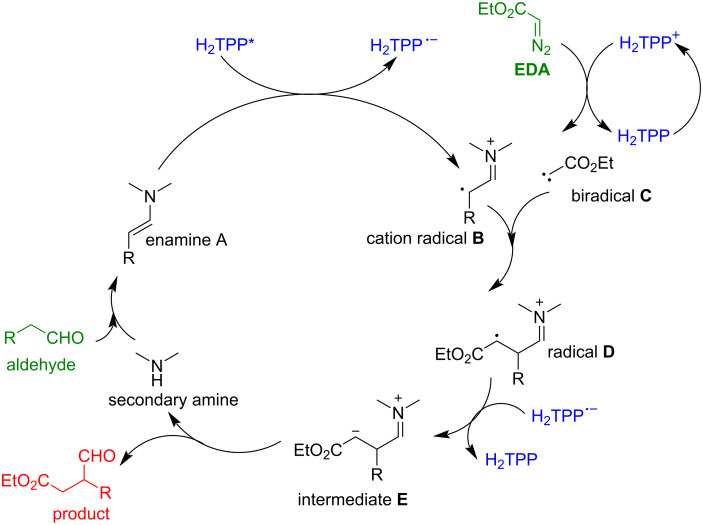
Proposed mechanism for the light-induced α-alkylation of aldehydes with EDA in the presence of H_2_TPP (**18**). Porphyrin acts as both photoredox catalyst and a photosensitizer. Adapted from [91].

Additionally, after light excitation, porphyrins can also oxidize a substrate by accepting electrons from a substrate molecule or transform into a long-lived radical cation by substrate reduction, which are the fundamentals of photoredox catalysis (Figure 13a). Monomeric porphyrins and supramolecular porous frameworks composed of porphyrin building blocks, such as metal-organic frameworks (MOF) and covalent organic frameworks (COF), have been extensively studied as photosensitizers of singlet oxygen and photoredox catalysts [87–90]. However, using metal-free porphyrins as photoredox catalysts for C–C or C–heteroatom bond formation is an area which has recently started to be explored. In 2016, Gryko and co-workers reported using metal-free planar H_2_TPP (**18**) as a photocatalyst for the photoredox-α-alkylation of aldehydes with ethyl diazoacetate [91]. This reaction achieved an impressive product yield up to 84%. Control experiments showed that omitting any one of the reaction components – such as the porphyrin catalyst, amine, aldehyde, EDA, or light source – completely halted the reaction, resulting in no product formation. The further study found that porphyrins with both electron-withdrawing and electron-donating substituents at the *meso*-positions were catalytically active. Additionally, among various amines tested, only the secondary amines (morpholine) led to product formation, confirming the formation of enamine in the catalytic cycle. The proposed mechanism suggested that the amine, photocatalyst, and light each played crucial roles (Figure 14). The porphyrin acted as both a photoredox unit and a photosensitizer, facilitating photoinduced electron transfer (PET) to form the active cation radical **B**, and intersystem crossing (ISC) for energy transfer to generate the triplet carbene **C**. Radical **B** then reacted with biradical **C**, producing the new radical **D**, which accepted an electron from the porphyrin radical anion. Ultimately, protonation of intermediate **E** led to the final product. Formation of intermediates, such as enamine **A** and cation radical **B**, was confirmed using techniques like ESIMS, ^1^H NMR, and EPR, Stern–Volmer quenching experiments, respectively. All these mechanistic studies suggested that the reaction of the porphyrin catalyst with the enamine and ethyl diazoacetate (EDA) played a crucial role in these α-alkylation reactions. This work demonstrated a dual catalytic system where porphyrin functioned as both a photoredox catalyst and a photosensitizer.

**Figure 15:**
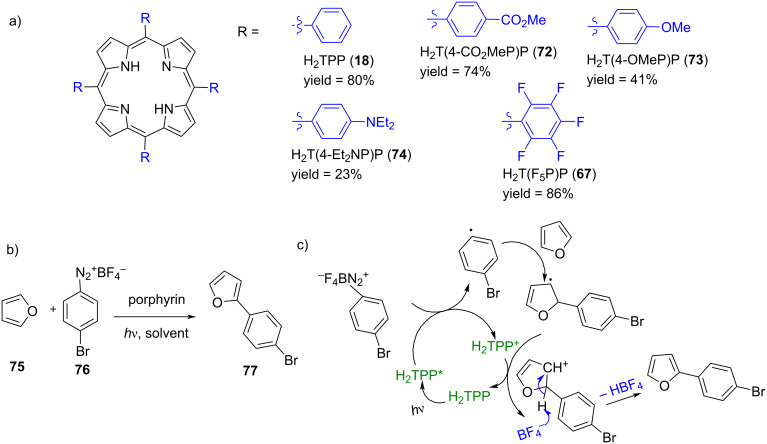
a) Chemical structures of porphyrins screened as photoredox catalysts, b) model reaction of furan (**75**) with 4-bromobenzenediazonium tetrafluoroborate (**76**), and c) proposed mechanism for the light-induced arylation of heteroarenes with diazonium salts. Adapted from [92].

Later, the same group used metal-free macrocycles for the C–H arylation of five-membered heteroarenes using aryldiazonium salts, with porphyrin serving as the photoredox catalyst [92]. Control experiments indicated that H_2_TPP (**18**), when irradiated with light, gave 80% yield of the C–H arylated product **77** for the reaction of furan (**75**) with 4-bromobenzenediazonium tetrafluoroborate (**76**) (Figure 15a and 15b). In contrast, negligible product (5%) was formed in the absence of light. When light was present but H_2_TPP (**18**) was absent, the yield was only 8%, likely due to light-triggered heterolysis of the diazonium salt, which initiated the reaction pathway. The authors proposed that under light irradiation, the porphyrin transitioned to its excited state, generating a phenyl radical through photoinduced single-electron transfer (Figure 15c). This phenyl radical then added to the furan (heteroarene), forming an aryl radical intermediate. This intermediate was subsequently oxidized by the porphyrin cation radical, leading to the formation of the final product and completing the catalytic cycle. They have further screened porphyrins with both electron-withdrawing and electron-donating groups at the periphery as potential photocatalysts. The results demonstrated that these substituents significantly influenced the redox properties of the porphyrins, yielding up to 86% with the electron-poor *meso*-tetrakis(pentafluorophenyl)porphyrin (**67**), compared to H_2_TPP and other electron-rich systems. This finding indicated that fine-tuning the electrochemical and photochemical properties of the catalyst was crucial for facilitating photoelectron transfer (PET) processes in these photoredox systems. De Oliveira and co-workers reported metal-free porphyrins as photoredox catalysts for the synthesis of α-arylketones/aldehydes by arylation of enol acetates with aryldiazonium salts [93]. The excitation of the porphyrin macrocycles by light irradiation initiated the catalytic cycle, generating aryl radicals from the diazonium salts, similar to findings by Gryko and co-workers. They explored both batch and continuous-flow photocatalysis using these systems, achieving improved yields of up to 92%. Notably, a multigram-scale experiment was successfully performed, producing 3.03 g of the desired product under continuous-flow conditions.

**Figure 16:**
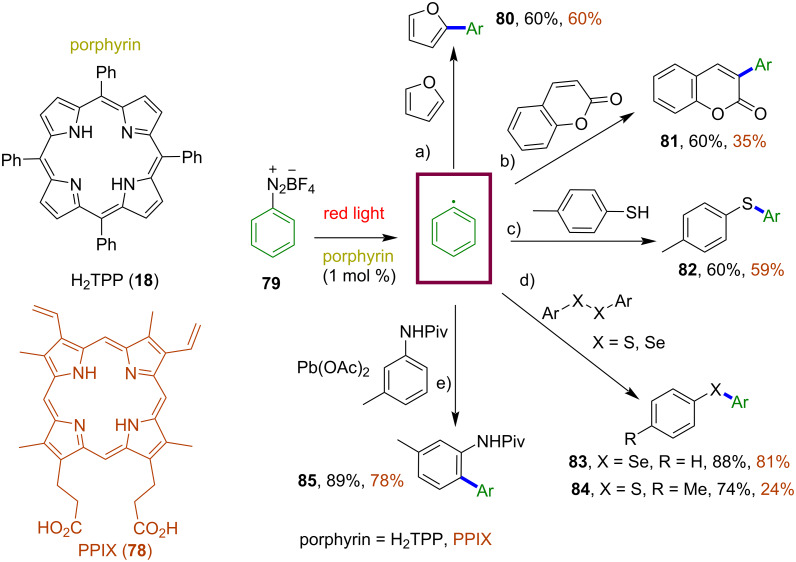
Porphyrin macrocycles H_2_TPP (**18**) and PPIX **78** as photoreductants for the red light-induced C–H arylation of a) furan, b) coumarin, c) thiol, d) ArXXAr (X = S, Se), and e) pivalamide. Adapted from [97].

**Figure 17:**
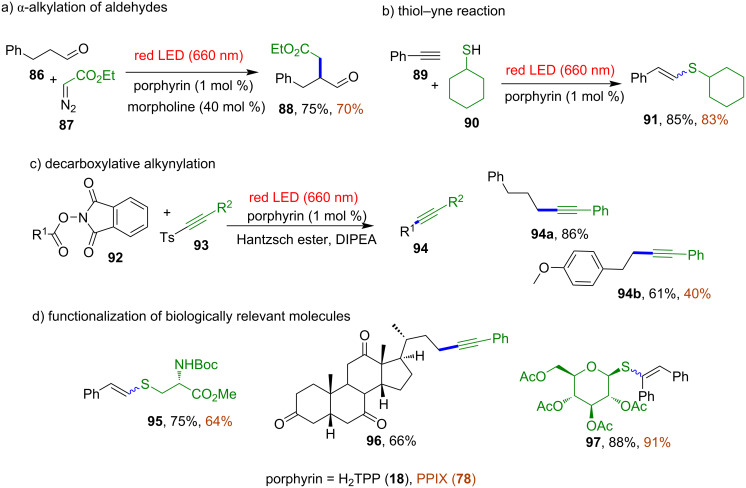
Porphyrin macrocycles H_2_TPP (**18**) and PPIX **78** as photoredox catalyst for (a) α-alkylation of an aldehyde, (b) thiol–yne reaction, (c) decarboxylative alkynylation, and (d) functionalization of biologically relevant molecules (d). Adapted from [97].

In 2020, de Oliveira and co-workers published a review covering the field of metal-free porphyrin macrocycles as photocatalysts in organic synthesis, involving both single electron transfer (SET) and energy transfer (ET) mechanistic approaches [84]. This review does not only focus on the metal-free porphyrin macrocycles, but it also covers the area of different porphyrinoid systems, such as heteroatom-containing macrocycles and metalloporphyrins. Despite the impressive progress in photoredox catalysis, due to their most intensive electronic absorption band at 420 nm (Soret band, extinction coefficient of 10^5^ M^−1^ cm^−1^), most porphyrin photocatalysts reported so far have been mainly utilized under blue light irradiation. There are a few reports on red light-mediated transformations using other pyrrolic macrocycles, such as thiaporphyrin [94], phthalocyanine [95], and subphthalocyanine [96]. Porphyrin macrocycles can also absorb red light (Q bands at 518, 553, 592, and 648 nm with extinction coefficients around 10^4^ M^−1^ cm^−1^), but they had not been used as photocatalysts in red light-induced processes until very recently. In 2022, Gryko and co-workers screened metal-free porphyrin macrocycles for various organic photochemical reactions that proceed via both oxidative and reductive quenching under red light irradiation [97]. Firstly, they evaluated the photoreductant role of metal-free macrocycles, H_2_TPP (**18**) and PPIX **78**, in the red light-induced C–H arylation of different substrates such as furan, coumarin, thiol, pivalamide, aryl thiaether and the selenium equivalents. Use of both macrocycles resulted in the formation of the product in 60–89% yields and 24–81% yields for **18** and **78**, respectively, confirming that even the less energetic red light is sufficient to generate aryl radicals via single-electron transfer (SET) from the excited porphyrin to aryldiazonium salt **79** (Figure 16). Further studies were focused on using these porphyrins as photooxidants in the red light-induced α-alkylation of aldehyde with ethyl diazoacetate. The reported reaction proceeds smoothly, giving 75% and 70% yields for macrocycles **18** and **78**, respectively (Figure 17a).

Irradiation of porphyrin photocatalysts by red light turned out to be as effective as blue light, providing good yields of various photochemical reactions that proceed via both oxidative and reductive quenching mechanisms. Further, using of metal-free porphyrins as photocatalysts in bioorthogonal chemistry was explored. They can be utilized in transformations of biomolecules, such as thiol–yne reaction and decarboxylative alkynylation. The thiol–yne reaction of cyclohexanethiol (**90**) with phenylacetylene (**89**) in the presence of 1 mol % of H_2_TPP (**18**) under red LED irradiation provided the desired product **91** in up to 85% yield while the decarboxylative alkynylation reaction of *N*-hydroxyphthalimide esters (NHPI) **92** with alkynyl *p*-tolylsulfones **93** in the presence of H_2_TPP (**18**) resulted in 44–93% yields depending on the substituents (Figure 17b and c).

Furthermore, the authors approved the biological application of porphyrin photoredox catalysts by using them in red light-induced C–X-bond formation on biologically relevant molecules **95**–**97**, based on a thiol–yne reaction and decarboxylative alkynylation protocol (Figure 17d). Last year, Moyano and colleagues reported on amino-functionalized porphyrins as bifunctional organophotocatalysts, effectively combining the organocatalytic and photocatalytic potential of porphyrin macrocycles [98].

**Figure 18:**
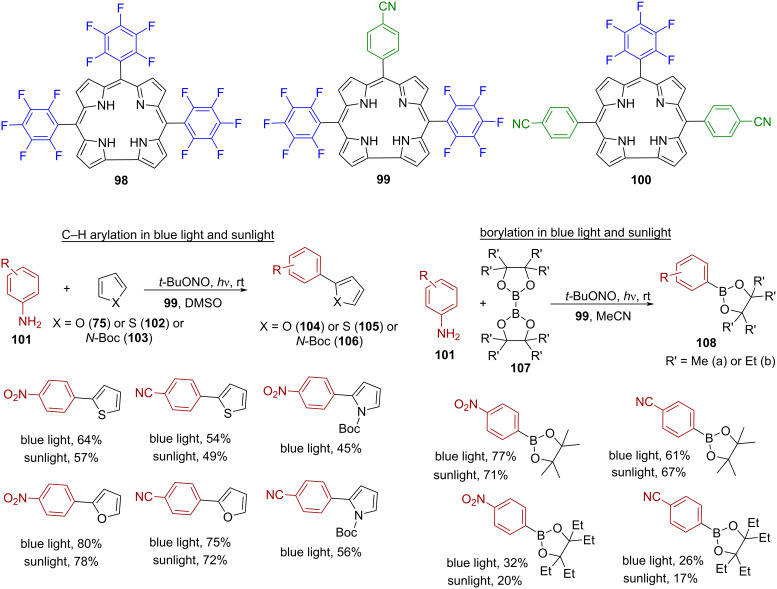
Corrole macrocycles **98**–**100** as photoredox catalysts for C–H arylation and borylation reactions. Adapted from [99].

In 2024, Gupta and colleagues expanded on the success of free base porphyrin macrocycles as photoredox catalysts by introducing *meso*-arylcorroles (types A_3_ and A_2_B) for C–H arylation and borylation reactions activated by sunlight [99]. This marked the first application of these corroles as photoredox catalysts. They synthesized three free base corroles **98**–**100** featuring electron-withdrawing substituents at the *meso*-positions and tested them for the arylation of furan (**75**), thiophene (**102**), and *N*-Boc-pyrrole (**103**) using substituted anilines **101** and *t*-BuONO (Figure 18). The reactions were conducted under light irradiation (blue light/sunlight) for 30 minutes in DMSO within an inert atmosphere. All corroles demonstrated catalytic activity with only 0.5% loading, while control experiments without a catalyst or light yielded minimal to no product. Among all the catalysts, corrole **99** turned out to be particularly effective in C–H-arylations, demonstrating high tolerance for various functional groups and higher product yields under both blue and sunlight. The authors suggested a radical mechanism similar to that of porphyrins, and provided evidence for aryl radical formation through mass spectrometry and NMR analysis of the adduct formed from the reaction between the radical intermediate and the scavenger 2,2,6,6-tetramethyl-1-piperidin-1-oxyl (TEMPO). Furthermore, they used the catalysts for borylation of arylamines **101**, using visible/sunlight to activate the catalyst (1 mol %) in acetonitrile with *t*-BuONO and B_2_pin_2_ (**107a**)/B_2_Epin_2_ (**107b**), achieving moderate to good yields of products ranging from 17% to 77%.

In general, porphyrin macrocycles, due to their 18-π-electron aromatic ring, small singlet–triplet splitting, high quantum yield for intersystem crossing, and long triplet state lifetime, act as robust electron mediators. This section highlights the use of metal-free porphyrins in organic photoredox catalysis. So far, mainly planar metal-free porphyrins have been explored as photoredox catalysts, paving the way for recent advancements, including the first example of photoredox catalysis using corroles. However, it would be interesting to see whether even β-functionalized metal-free porphyrins or other tetrapyrrolic macrocycles can be used in photoredox transformations.

### 3 Metal-free tetrapyrrolic macrocycles as electrocatalysts

Development of efficient renewable technologies is a driving force in the efforts to achieve sustainability with the same or even increasing demands for energy worldwide. In this context, transition-metal complexes of tetraazamacrocycles (N_4_-macrocycle) such as porphyrins, cyclam (tetraazacyclotetradecane), phthalocyanines, corroles and their supramolecular frameworks have been widely used as both homogeneous and heterogeneous electrocatalysts for various energy conversion and storage techniques, such as fuel cells, water splitting devices, and rechargeable metal–air batteries, due to the ease of their structural modification, rich redox chemistry, and robust coordination M–N_4_ environment [100–105]. The key processes employed in energy transfer and storage are the oxygen reduction reaction (ORR), hydrogen evolution reaction (HER), and oxygen evolution reaction (OER). There are several reviews focusing on the relationship between metallo-catalyst structures and HER, OER, and ORR performance/mechanisms, selection of the central metal ion, and peripheral functionalization of the catalysts [106–109]. This review summarizes recent achievements in the catalysis of ORR, HER, and OER processes using metal-free porphyrin macrocycles.

Similarly to their metallated counterparts, metal-free porphyrin macrocycles can also act as electrocatalyst for HER, OER, and ORR processes [110–119]. In the case of metalloporphyrin catalysts, the metal center acts as a catalytic site, whereas in the case of metal-free macrocycles, different mechanistic routes have to be followed, as explained later in this review. First, reported examples of metal-free porphyrin macrocycles used as electrocatalysts for HER reactions will be summarized.

**Figure 19:**
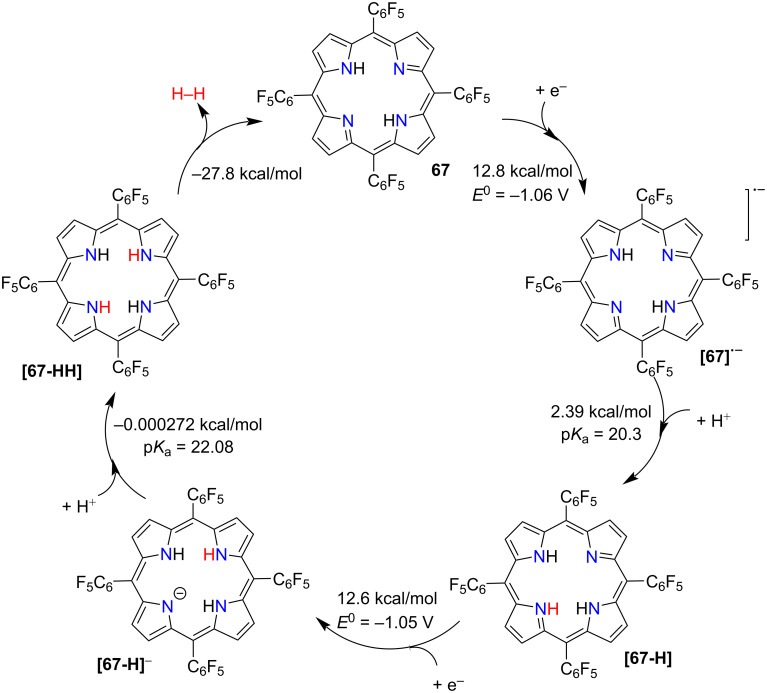
Proposed catalytic cycle of electrocatalytic generation of H_2_ evolution using tetrapyrrolic macrocycle **67** (following an E–P–E–P mechanism). Adapted from [111].

In 2014, Kadish and co-workers reported a series of planar and nonplanar metal-free tetraarylporphyrins, indicating the potential of these macrocycles for the generation of molecular hydrogen under acidic conditions [110]. Four years later, Villagrán and co-workers used electron-deficient metal-free *meso*-tetra(pentafluorophenyl)porphyrin (**67**) as a HER electrocatalyst using TsOH (*p*-toluenesulfonic acid) as a proton donor in THF [111]. Macrocycle **67** undergoes two reversible one-electron reductions at *E*_1/2_ = −1.14 V and −1.54 V yielding radical anion [**67**]˙^−^ and a dianion species [**67**]^2−^. Upon increasing addition of TsOH into **67**, an increase in electrocatalytic current appeared before the second reduction wave, while the first reduction at −1.14 V remained unchanged, suggesting formation of radical anion [**67**]˙^−^ as the first step. UV–vis spectroelectrochemical measurements under bulk electrolysis conditions also supported the generation of the radical anion [**67**]˙^−^. Furthermore, the authors used thermodynamic theoretical calculations to investigate catalytic steps, finding that the protonation of [**67**]˙^−^ to generate [**67-H**] is thermodynamically favored (free energy of +2.39 kcal mol^−1^) over its reduction to highly energetic dianion [**67**]^2−^ (free energy of +36.3 kcal mol^−1^). Combining experimental and theoretical observations, the authors proposed the most favorable hydrogen generation mechanism to be E–P–E–P; where E stands for reduction and P means protonation (Figure 19). Acid protonates the radical anion [**67**]˙^−^ to give [**67-H**], the following reduction leads to thermodynamically favored [**67-H**]^−^, which then undergoes protonation and yields [**67-HH**]. In the last step, [**67-HH**] produces H_2_, and closes the catalytic cycle. Later, a different type of a metal-free tetrapyrrolic macrocycle, corrole, was used for controlling the electrocatalyzed H_2_ evolution in acidic conditions in acetonitrile [112].

There are not many reported examples of metal-free porphyrin macrocycles as HER electrocatalysts. Metal-free porphyrin macrocycles are capable of multielectron redox processes and have basic amine nitrogen atoms that can form nitrogen–hydrogen (N–H) bonds. Therefore, the general mechanism for HER using a metal-free porphyrin involves inner core nitrogen atoms, which keep protons in proximity and lowers the activation barrier. Dihydrogen is then produced by prearranging the transition state of hydrogen–hydrogen (H–H) bond formation. This mechanism differs from metalloporphyrins, where both the metal and the ligand are redox-active [120]. Considering the potential of metal-free porphyrins as promising electrocatalysts, researchers have also investigated similar macrocycles, such as corroles, for hydrogen evolution reactions (HER). While metal corroles have been extensively studied as efficient electrocatalysts [100,121–122], no reports on metal-free corroles were available until 2020. Si and co-workers reported that cobalt and metal-free triarylcorroles bearing hydroxyethylamino groups exhibited activity in electrocatalytic HER [123]. Although free base corrole ligands demonstrated activity in HER, they were unstable in trifluoroacetic acid (TFA), a common proton source, leading to rapid degradation during catalysis. Subsequently, the same group introduced metal-free xanthene-bridged biscorroles and tested them as HER electrocatalysts using acetic acid as an alternative proton source [124]. Preliminary results indicated that the biscorrole (1.5 mg, 1 μM) could produce 0.84 mL of H₂ during 1 hour of electrolysis, as confirmed by gas chromatography (GC). Villagrán and co-workers reported a combination of computational and experimental methods to study the electrocatalytic activity of the hydrogen evolution reaction (HER) catalyzed by free base 5,10,15-tris(pentafluorophenyl)corrole (**98**) [112]. Their work showed that using *p*-toluenesulfonic acid as the proton source, **98** was able to produce hydrogen (H_2_) electrochemically in acetonitrile, although their proposed mechanism for HER was different from the metal-free porphyrin macrocycles. Compared to metal-free porphyrins, the research on corroles as electrocatalysts is still in its early stages. However, these reports highlight the potential of corroles for future advancements in this area.

The following section reports advancements in the field of oxygen reduction reactions (ORR), also known as oxygen electrocatalysis, using metal-free porphyrin macrocycles as electrocatalysts. ORR is an important biological process, as Fe-porphyrin heme sites activate and reduce O_2_ [125]. Inspired by this process, many synthetic Fe-porphyrins and related metal macrocycles have been designed and investigated as catalysts for O_2_ reduction. In catalytic oxygen reduction reactions (ORR) involving metalloporphyrins, it is typically suggested that O_2_ initially binds to the vacant axial site of the metal centers. This binding is followed by reduction to either hydrogen peroxide (H_2_O_2_) via a two-electron (2e^−^) pathway, water (H_2_O) through a four-electron (4e^−^) pathway, or a combination of both products through a concerted transfer of electrons and protons [126]. The specific catalyst employed significantly influences catalytic activity, long-term stability, and product selectivity, especially regarding the characteristics of the active metal sites and the functional substituents on the macrocycle.

**Figure 20:**
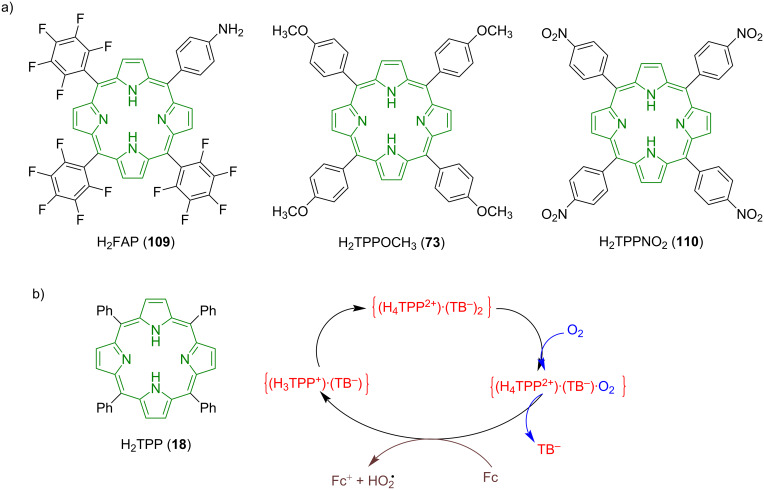
a) Chemical structures of tetrapyrrolic macrocycles **109**, **73**, and **110** used for oxygen reductions in one- and two-phase liquid systems; b) catalytic cycle for reduction of O_2_ to HO_2_˙ using diprotonated porphyrin **18^2+^** . Adapted from [117–119].

Considering the successful use of metalloporphyrins for the reduction of O_2_ to H_2_O_2_ and/or to H_2_O, Samec and co-workers have done a substantial amount of work exploring the use of metal-free porphyrins as catalysts for ORR [113–118]. They reported that metal-free porphyrin macrocycles; **109** (5-(*p*-aminophenyl)-10,15,20-tris(pentafluorophenyl)porphyrin, H_2_FAP), and **18** (5,10,15,20-*meso*-tetraphenylporphyrin, H_2_TPP) can catalyze oxygen reduction to H_2_O_2_ using ferrocene-based electron donors [ferrocene (Fc) and decamethylferrocene (DMFc)] at acidified water/1,2-dichloroethane (DCE) interface [115,117]. This two-phasic oxygen reduction undergoes via binding of O_2_ to diprotonated forms of porphyrins, **109^2+^** (H_4_FAP^2+^) and **18^2+^** (H_4_TPP^2+^), which are then reduced in the organic phase by ferrocene-based reductants, resulting in H_2_O_2_, Fc^+^/DMFc^+^, and the respective metal-free porphyrin macrocycle (Figure 20a and b). The conditions of homogeneous O_2_ reduction were further explored using **18** (H_2_TPP) as a catalyst, Fc as an external reductant, DCE as a solvent, and two different compounds as proton sources: tetrakis(pentafluorophenyl)boric acid (HTB) and trifluoroacetic acid (TFA) [118].

**Figure 21:**
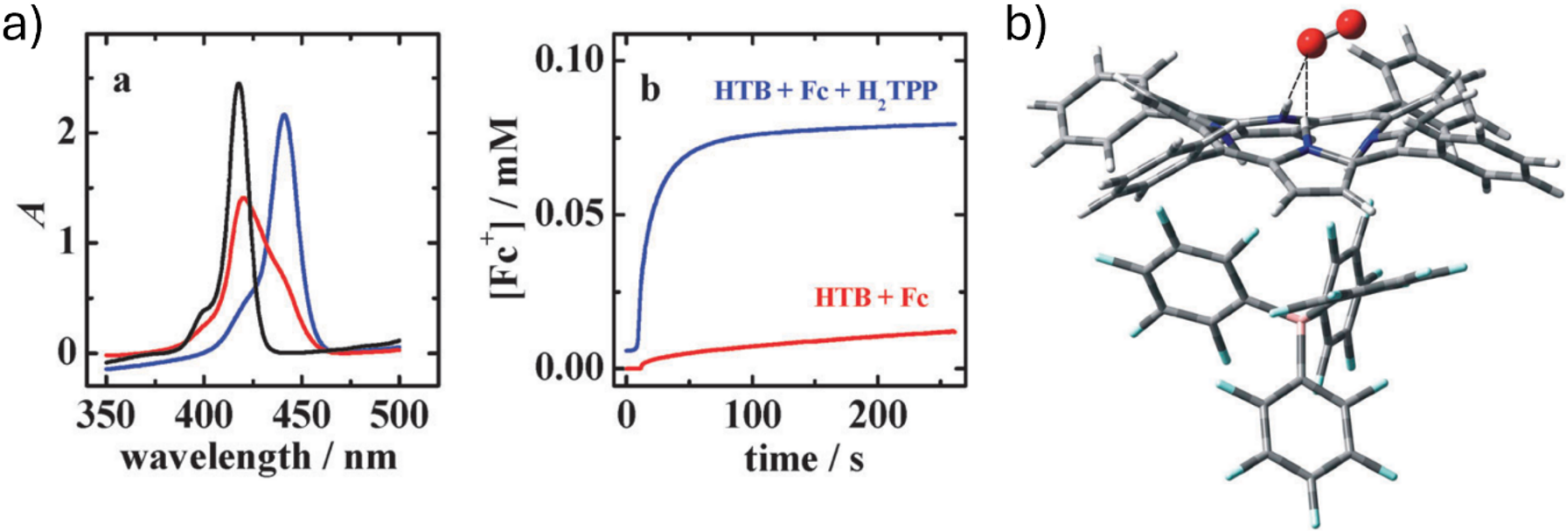
a) Absorption spectra (left) of the air-saturated DCE solutions containing: 5 × 10^−5^ M H_2_TPP (black line), 5 × 10^−5^ M H_2_TPP, and 5 × 10^−5^ M HTB (red line) or 1.25 × 10^−4^ M HTB (blue line), and a time profile (right) of conversion of Fc to Fc^+^ monitored at 300 nm; b) DFT-optimized structure of {(H_4_TPP^2+^)·(TB^−^)·O_2_} system. Used with permission of the Royal Society of Chemistry, from [118] (“Fine tuning of the catalytic effect of a metal-free porphyrin on the homogeneous oxygenreduction” by A. Trojánek et al, Chem. Commun., vol. 47, issue 19, © 2011); permission conveyed through Copyright Clearance Center, Inc. This content is not subject to CC BY 4.0.

After HTB was added to **18** (H_2_TPP) in 1:2.5 molar ratio, the Soret band in the UV–vis spectrum revealed a red shift, indicating the presence of diprotonated H_4_TPP^2+^ macrocycle (Figure 21a). Fc addition into an air-saturated DCE solution containing **18** (H_2_TPP) and HTB led to oxidation of Fc to Fc^+^ and initiated the ORR process (Figure 20b). The rate of ferrocene oxidation (Fc to Fc^+^) was reported to be very slow and independent of HTB concentration in the absence of **18** (H_2_TPP), suggesting that the porphyrin macrocycle is necessary for the O_2_ reduction to H_2_O_2_ (Figure 21a). From these observations, the authors concluded that O_2_ binding to the diprotonated form of **18** (H_2_TPP) via NH^+^···O_2_ hydrogen bonds initiated the ORR, whereas an increase in the HTB concentration inhibited the ORR by blocking NH^+^ binding sites for O_2_. Further, the role of the proton source on ORR was confirmed by testing a stronger acid, TFA: in this case, the O_2_ reduction rate is decreased to almost zero due to too strong association of trifluoroacetate with protonated porphyrin. DFT calculations suggested that the O–O bond in O_2_ becomes polarized upon binding in {(H_4_TPP^2+^)·(TB^−^)·O_2_}, which facilitates the activation of O_2_, similarly to metal porphyrins (Figure 21b).

After the successful O_2_ reduction by diprotonated porphyrins, the inhibitory effect of H_2_O on catalytic ORR by **18** (H_2_TPP) was studied using UV–vis absorption, electrochemical methods, and DFT calculations [117]. The reported rate of conversion of Fc to Fc^+^ in the presence of an air-saturated DCE solution containing O_2_, HTB, and the porphyrin macrocycle decreased sharply with the increasing water concentration. The decrease was attributed to the concurrence of H_2_O molecules to O_2_ in formation of the complex with protonated porphyrin.

Su and co-workers followed up the previous work by a study of the effect of electron-deficient and electron-rich tetrapyrrole macrocycles on ORR [119]. They used three metal-free porphyrins with different electron-withdrawing and electron-donating functionalities at *meso*-position (Figure 20); **18** (H_2_TPP), **73** (5,10,15,20-tetrakis(4-methoxyphenyl)porphyrin, H_2_TPPOCH_3_), and **110** (5,10,15,20-tetra(4-nitrophenyl)porphyrin, H_2_TPPNO_2_) towards oxygen reduction by ferrocene (Fc) and 1,1’-dimethylferrocene (DFc) at the water–DCE interface. As the reduction of O_2_ to H_2_O_2_ is initiated by binding of O_2_ to –NH^+^ sites of a diprotonated porphyrin macrocycle, the reaction is affected mainly by the ease of the macrocycle protonation. ORR studies showed that all the three macrocycles were catalytically active and their activity followed the trend **73** > **18** > **110**, as the macrocycle **73**, with electron-donating *meso*-substituents gets protonated more easily than **18**, whereas **110** with *meso*-nitrophenyl groups is harder to be protonated. Hence, ORR was reported to work better on electron-rich macrocycles than on electron-deficient ones.

**Figure 22:**
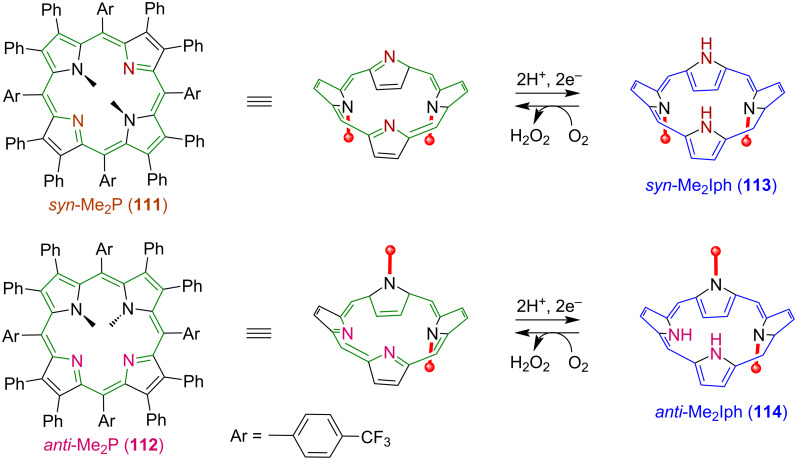
Chemical structures of *N*,*N*’-dimethylated saddle-distorted porphyrin isomers, syn-Me_2_P **111** and anti-Me_2_P **112** as electrocatalysts of ORR forming H_2_O_2_. Adapted from [127].

**Figure 23:**
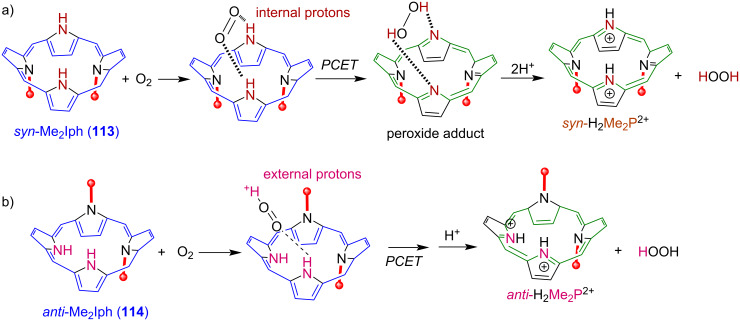
Reaction mechanisms for the two-electron reduction of O_2_ by a) *syn*-Me_2_Iph **113** and b) *anti*-Me_2_Iph **114**. Adapted from [127].

Samec and co-workers’ study of ORR is based on the use of planar porphyrin macrocycles as electrocatalysts, that become nonplanar once diprotonated. This nonplanar diprotonated porphyrin with accessible inner NH groups activates O_2_. Later, to study the effect of nonplanarity or distortion of macrocyclic core on evaluation of ORR reactivity, Kojima and co-workers synthesized two isomers of *N*,*N*’-dimethylated saddle-distorted porphyrin, *syn*-Me_2_P **111** and *anti*-Me_2_P **112**, and used them as catalysts for two-electron-reduction of O_2_ to H_2_O_2_ in the presence of Me_8_Fc (octamethyl ferrocene) as an electron donor and TFA (trifluoroacetic acid) as a proton source (Figure 22) [127]. The reported turnover number (TON) of H_2_O_2_ production was 32 with 64% yield for *syn*-Me_2_P **111**, whereas higher TON of 50 with 100% yield was observed for *anti*-Me_2_P **112**, and no electrocatalysis was observed in the absence of macrocyclic catalysts. Both *syn*-Me_2_P **111** and *anti*-Me_2_P **112** macrocycles formed diprotonated species (*syn*-H_2_Me_2_P^2+^ and *anti*-H_2_Me_2_P^2+^) in the presence of TFA, which were then reduced to isophlorins *syn*-Me_2_Iph **113** and *anti*-Me_2_Iph **114** with Me_8_Fc as a reductant. These two-electron-reduced isophlorin species, *syn*-Me_2_Iph **113** and *anti*-Me_2_Iph **114**, act as reaction intermediates. Kinetic analysis showed that the rate of formation of Me_8_Fc^+^ from Me_8_Fc is independent of the concentration of acid. The authors proposed that *syn*-Me_2_Iph **113** forms a two-point hydrogen bonding to O_2_ and reduces O_2_ to H_2_O_2_ through proton-coupled electron transfer (PCET), whereas *anti*-Me_2_Iph **114** forms only a one-point hydrogen bonding to O_2_ that picks up one external proton producing H_2_O_2_ and the protonated porphyrin macrocycle (*anti*-H_2_Me_2_P^2+^) (Figure 23).

**Figure 24:**
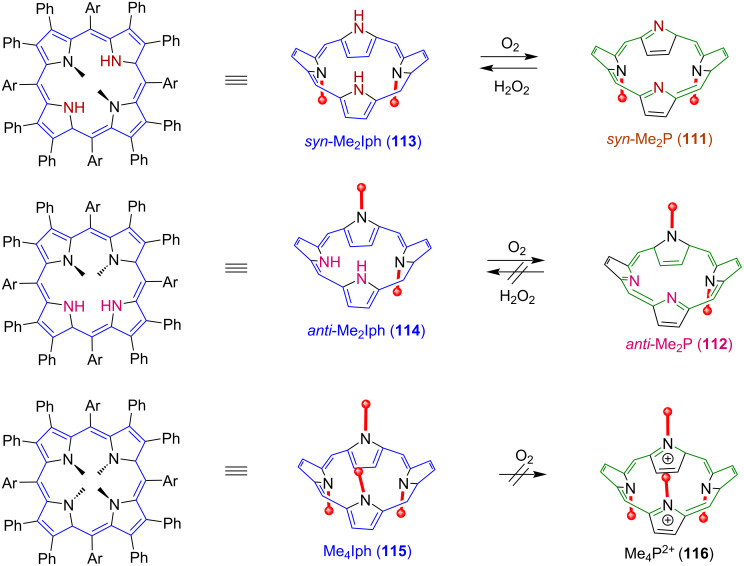
O_2_/H_2_O_2_ interconversion using methylated saddle-distorted porphyrin and isophlorin (reduced porphyrin) macrocycles **111**–**116**. Adapted from [128].

Later, O_2_/H_2_O_2_ interconversion using dimethylated saddle-distorted porphyrin and isophlorin (reduced porphyrin) macrocycles **111** and **112** [128] was reported. The *N*_21_,*N*_23_-dimethylated isophlorin (*syn*-Me_2_Iph) **113** macrocycle binds with O_2_ and results in ORR forming H_2_O_2_ as a product following the same mechanism as discussed above. The interconversion between **111** and **113** is reversible and **111** can be transformed back to **113** following the oxidation of H_2_O_2_ (Figure 24).

**Figure 25:**
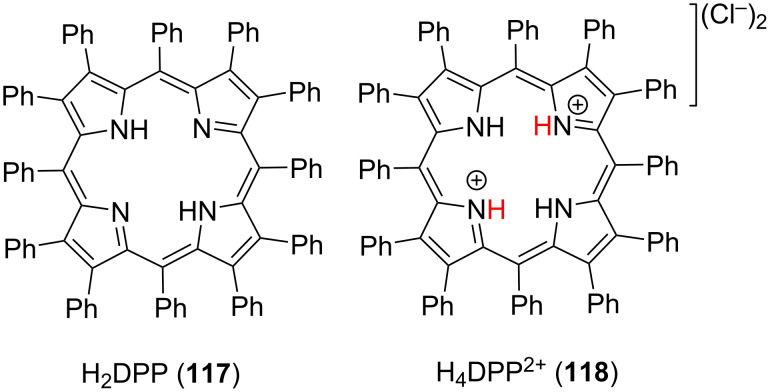
Chemical structures of distorted dodecaphenylporphyrin macrocycle **117** and its diprotonated form **118** used as photocatalysts for O_2_ reduction and H_2_ evolution. Adapted from [129–130].

A related tetraalkylated isophlorin **115** (Me_4_Iph) macrocycle cannot be transformed to its porphyrin analogue by O_2_, showing the importance of accessible inner –NHs for ORR, while the *N*_21_,*N*_22_-dimethylated porphyrin **112** (*anti*-Me_2_P), which lacks the multipoint hydrogen-bonding sites for H_2_O_2_, does not undergo reduction to the corresponding isophlorin, supporting the importance of hydrogen-bonding interactions to achieve the O_2_/H_2_O_2_ interconversion (Figure 24). This interconversion happens due to the appropriate arrangement of inner –NH protons in the isophlorin core forming hydrogen bonding with O_2_ as well as those of the lone pairs of the inner nitrogen atoms forming hydrogen bonding with H_2_O_2_. They also successfully used saddle-distorted dodecaphenylporphyrin **117** (H_2_DPP) and its diprotonated form H_4_DPP^2+^** 118** as a photocatalyst for oxygen reduction to H_2_O_2_ (Figure 25) [129]. H_4_DPP^2+^ (**118**), upon photoexcitation in the presence of an electron donor (10-methyl-9,10-dihydroacridine, AcrH_2_), generates H_4_DPP˙^+^ via photoinduced electron transfer (ET). Further, proton-coupled electron transfer (PCET) from H_4_DPP˙^+^ to O_2_, in the presence of a proton source, results in efficient photocatalytic activity for H_2_O_2_ production. Later, it was found out that the H_4_DPP^2+^ (**118**) macrocycle can also act as a photocatalyst for the hydrogen (H_2_) evolution reaction in the presence of poly(vinylpyrrolidone)-protected PtNPs, where 10-methyl-9,10-dihydroacridine (AcrH_2_) acts as a two-electron donor and *p*-toluenesulfonic acid (TsOH) as a proton source [130]. The mechanistic studies suggested that the mechanism of H_2_ evolution consists of a photoinduced ET from AcrH_2_ to excited H_4_DPP^2+^ providing H_4_DPP^•+^, followed by an electron injection directly from H_4_DPP^•+^ to PtNPs reducing a proton. The formed putative Pt–H species on the surface of PtNPs is then decomposed with evolution of H_2_.

As compared to HER and ORR, electrochemical water splitting and OER using porphyrins have been mainly done by using metalloporphyrins [131–133] where a nucleophilic attack of water or hydroxide on high-valent metal metal-oxo intermediates results in O–O-bond formation. There are no examples of using a metal-free porphyrin as an electrocatalyst for OER, although their use for HER and ORR electrocatalysts makes them potential future catalysts for various energy conversion and storage techniques.

## Conclusion

This review is mainly focused on metal-free tetrapyrrolic macrocycles acting as catalysts. Both calix[4]pyrroles and porphyrins have been studied as metal-free catalytic systems in organic synthesis, particularly in organocatalysis. The conformational flexibility of calix[4]pyrrole macrocycles usually leads to less defined microenvironments for catalysis, despite the easy accessibility of the inner –NHs for substrate binding and activation. As only simple unfunctionalized calix[4]pyrrole macrocycles have been used as catalysts so far, there is a possible direction to explore the use of conformationally rigid (strapped-, capped- and bis-calix[4]pyrrole) skeletons. In contrast to calix[4]pyrroles, the exploration of metal-free porphyrins as organocatalysts has started on very recently. Emphasizing the nonplanarity/distortion of tetrapyrrolic cores is necessary to achieve catalytic activity, but later work in this field (using amphiphilic porphyrins and porphyrins with co-catalysts) has proven that even planar porphyrins could act as organocatalysts. In addition to organocatalysis, porphyrins have also been used as both photocatalysts and electrocatalysts due to their rich redox chemistry and photosensitizing properties. Compared to their metalloporphyrin counterparts, there are less reports, but nonetheless they have shown promising results, particularly in red light-induced photoredox catalysis as well as for HER and ORR processes. Although the field of synthetic porphyrin chemistry has been studied over many decades, using metal-free macrocycles as catalysts has only recently started providing promising results. Considering these, the feasibility of different catalytic outcomes and the already established synthetic methodologies, both calix[4]pyrroles and metal-free porphyrins are excellent candidates for catalysis. In addition to these two types of macrocycles, other pyrrolic macrocycles such as corroles, phthalocyanines and related systems can be also explored as potential catalysts.

## Data Availability

Data sharing is not applicable as no new data was generated or analyzed in this study.
